# Interventions Aimed at Enhancing Health Care Providers’ Behavior Toward the Prescription of Mobile Health Apps: Systematic Review

**DOI:** 10.2196/43561

**Published:** 2023-02-27

**Authors:** Ohoud Alkhaldi, Brian McMillan, Noha Maddah, John Ainsworth

**Affiliations:** 1 Division of Informatics, Imaging and Data Sciences, School of Health Sciences Faculty of Biology, Medicine and Health The University of Manchester Manchester United Kingdom; 2 Health Information Management and Technology Department College of Public Health Imam Abdulrahman Bin Faisal University Dammam Saudi Arabia; 3 Centre for Primary Care and Health Services Research The University of Manchester Manchester United Kingdom; 4 Department of Health Services and Hospitals Administration Faculty of Economics and Administration King Abdulaziz University Jeddah Saudi Arabia

**Keywords:** mHealth, mobile apps, prescription, behavioral change, mobile phone

## Abstract

**Background:**

Mobile health (mHealth) apps have great potential to support the management of chronic conditions. Despite widespread acceptance of mHealth apps by the public, health care providers (HCPs) are reluctant to prescribe or recommend such apps to their patients.

**Objective:**

This study aimed to classify and evaluate interventions aimed at encouraging HCPs to prescribe mHealth apps.

**Methods:**

A systematic literature search was conducted to identify studies published from January 1, 2008, to August 5, 2022, using 4 electronic databases: MEDLINE, Scopus, CINAHL, and PsycINFO. We included studies that evaluated interventions encouraging HCPs to prescribe mHealth apps. Two review authors independently assessed the eligibility of the studies. The “National Institute of Health’s quality assessment tool for before-and-after (pretest-posttest design) studies with no control group” and “the mixed methods appraisal tool (MMAT)” were used to assess the methodological quality. Owing to high levels of heterogeneity between interventions, measures of practice change, specialties of HCPs, and modes of delivery, we conducted a qualitative analysis. We adopted the behavior change wheel as a framework for classifying the included interventions according to intervention functions.

**Results:**

In total, 11 studies were included in this review. Most of the studies reported positive findings, with improvements in a number of outcomes, including increased knowledge of mHealth apps among clinicians, improved self-efficacy or confidence in prescribing, and an increased number of mHealth app prescriptions. On the basis of the behavior change wheel, 9 studies reported elements of environmental restructuring such as providing HCPs with lists of apps, technological systems, time, and resources. Furthermore, 9 studies included elements of education, particularly workshops, class lectures, individual sessions with HCPs, videos, or toolkits. Furthermore, training was incorporated in 8 studies using case studies or scenarios or app appraisal tools. Coercion and restriction were not reported in any of the interventions included. The quality of the studies was high in relation to the clarity of aims, interventions, and outcomes but weaker in terms of sample size, power calculations, and duration of follow-up.

**Conclusions:**

This study identified interventions to encourage app prescriptions by HCPs. Recommendations for future research should consider previously unexplored intervention functions such as restrictions and coercion. The findings of this review can help inform mHealth providers and policy makers regarding the key intervention strategies impacting mHealth prescriptions and assist them in making informed decisions to encourage this adoption.

## Introduction

### Background

The number of patients living with chronic conditions continues to increase worldwide [1], and empowering these patients to manage their diseases is vital. Mobile health (mHealth) provides digital solutions to patients to help them track and manage their diseases. With the increased number of available mHealth apps to download and use [2], it is expected that the number of consumers, whether they are members of the general public, patients, or health care providers (HCPs), will continue to grow. The purpose of different types of mHealth apps vary from well-being, prevention, management, and monitoring to follow up with HCPs. Some of these apps may be potentially suitable to be prescribed to patients for the diagnosis or treatment of medical conditions. The concept of “prescribable” health apps is recently introduced to refer to health apps that are currently available and have demonstrated effectiveness [3].

Studies evaluating the effectiveness of mobile apps on health outcomes are increasing in number. Several systematic reviews have concluded that mobile apps have the potential to improve patients’ health conditions, such as diabetes [4], mental health [5], and cardiovascular diseases [6]. Governments in several countries have acknowledged the benefits of mHealth and have endeavored to meet the urgent need to accelerate the adoption of mHealth apps. Germany became the first country in the world to prescribe mobile apps. HCPs can prescribe mHealth apps, which can be reimbursed by health insurance companies [7]. In the United Kingdom, the National Institute for Health and Care Excellence has published guidance about “Sleepio,” a digital therapeutic to treat insomnia, and has recommended the use of Sleepio as a cost-saving option in comparison with sleep hygiene or sleeping pills [8]. On the basis of the results of 28 studies, it has been concluded that Sleepio is more effective than the usual treatment in reducing symptoms of insomnia in adults [9].

In a survey study, HCPs from the United Kingdom were more likely to prescribe apps if they were tagged with National Health Service approval or recommended by work colleagues [10]. The same study reported that National Health Service–approved mHealth apps were more influential than evidence-based research. In Germany, a study of physicians’ attitudes toward prescribable mHealth apps found that only one-third of the physicians intended to prescribe apps, and the rate of HCPs who had already prescribed them was lower than expected, despite the existence of regulations and facilitators from the government accelerating the mHealth app adoption among HCPs [11]. The study authors suggested that a range of factors influenced app prescribing, including gender, age, the lack of intention to prescribe, and limited apps for some specialties.

These studies shed light on various barriers to mHealth app adoption in clinical care and provide opportunities to design future behavior change interventions to improve HCPs’ evidence-based app prescription behaviors. To date, there have been no systematic or comprehensive literature reviews that compile evidence of interventions for enhancing HCPs’ app prescription behaviors. Bringing together the findings from such interventions could potentially provide policy makers and stakeholders with a better understanding of valuable strategies that can be implemented to enhance HCPs’ uptake of mHealth apps. In this review, we address this gap by identifying interventions that aim to redirect HCPs’ behavior toward prescribing or recommending apps to patients.

### Behavior Change Framework

Several approaches are available to guide behavior change intervention designs. Among these approaches are the person-based approach [12], the British Medical Research Council’s framework on the development and evaluation of complex interventions [13], and intervention mapping [14]. Although each of these approaches offers considerable value to researchers, each concentrates on a different component of intervention development or has been criticized for lacking comprehensiveness and coherence [15].

**Figure 1:**
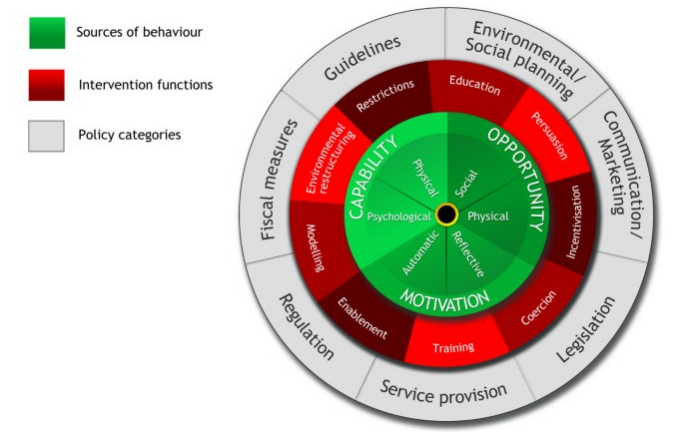
The behaviour change wheel (reproduced from Michie et al et al[15], which is published under Creative Commons Attribution 4.0 International License [17]).

The behavior change wheel (BCW) is used to characterize and evaluate behavior change interventions [15]. This framework provides a comprehensive approach to identifying sources of behavior and classifying them into the capability, opportunity, motivation, and behavior (COM-B) model, which represents the wheel’s hub (Figure 1). These components interact with each other to produce a change in behavior. Surrounding this is a layer of 9 intervention functions that can be selected depending on the behavioral analysis reached with the COM-B. The final layer contains 7 types of policies that one can use to deliver these intervention functions. The intervention functions are connected to behavior change techniques, which are the smallest active elements of an intervention (eg, self-monitoring, goal setting, action planning etc) [16]. Behavior change techniques used in interventions can be categorized using a taxonomy comprising 93 different techniques.

Understanding the target behavior is essential before designing an intervention. However, the BCW can also be applied retrospectively to intervention studies to identify and describe the behavior change strategies that have been used. It can also be used to improve current interventions or to introduce and evaluate an intervention that looks promising. Therefore, the interventions included in this review are classified into intervention functions of the BCW.

### Objectives

The study objectives were (1) to summarize and evaluate interventions aimed at encouraging HCPs to prescribe mHealth apps to patients and (2) to classify and map intervention strategies with the intervention functions of the BCW.

## Methods

### Research Question

This study was based on the PRISMA (Preferred Reporting Items for Systematic Reviews and Meta-analyses) guidelines [18]. The research question was created based on the PICO framework (population, intervention, comparator, and outcome) and was defined as follows: “How do interventions designed to encourage mobile health app prescription change the practices, knowledge or self-confidence of healthcare providers?”

### Eligibility Criteria

The population of interest was HCPs or trainees (eg, health personnel, general practitioners [GPs], physicians, clinicians, dietitians, and students of health specialties). Intervention studies to encourage mHealth app prescriptions, regardless of the design, were considered eligible. The primary outcome of interest reflected any measures of practice or behavior changes such as number of app prescriptions or self-reported or objectively measured changes in knowledge or confidence. The included studies had to be conducted in primary care settings. Studies were excluded if they were about patients’ adoption of mHealth apps or interventions to improve patients’ health outcomes. Other mHealth technologies such as wearables, mobile phone messaging, video consultations, or electronic health records (EHRs) were excluded. Studies of apps for HCPs’ medical education and training or decision support systems via mobile devices were also excluded.

### Sources of Information and Search

A search of 4 electronic databases (MEDLINE, Scopus, CINAHL, and PsycINFO) was conducted to identify studies published between January 1, 2008, and August 5, 2022. The official start of mobile apps was chosen as 2008, as the iPhone App Store was launched that year [19]. Studies published in English and peer-reviewed papers were included. A manual search of the reference lists of eligible studies was conducted. Medical Subject Headings terms were used wherever possible to locate the relevant studies. The Boolean operators AND and OR were used to enhance the search strategy. The search string used in 2 databases is shown in Multimedia Appendix 1.

### Study Selection

The search results were exported to the EndNote Web software (Clarivate Analytics) for screening and removing duplicates. After eliminating duplicates, screening of all titles and abstracts was independently conducted by 2 reviewers (OA and NM). The same 2 researchers reviewed the full texts of the papers identified as relevant to the objectives. In cases of disagreement, the research team discussed them and made the final decision.

### Risk of Bias Assessment

The risk of bias was assessed using 2 quality appraisal tools based on the study design. The National Institute of Health’s quality assessment tool for before-and-after (pretest-posttest design) studies with no control group was used for uncontrolled before-and-after studies [20]. This tool is composed of 12 items with response options “yes,” “no,” “not reported,” and “not applicable,” and the overall quality of each study can be classified as “good,” “fair,” or “poor.” The grading was decided by the total score: 0 to 4 (poor), 5 to 9 (fair), and 10 to 12 (good).

The mixed methods appraisal tool (MMAT) was used to assess the quality of the remaining studies. The MMAT is a comprehensive tool for assessing the quality of quantitative, qualitative, and mixed methods study designs [21]. This tool begins with 2 screening questions to determine whether a research objective is clear and whether the collected data allow a research question to be answered. The remaining 5 questions assess the methodologies. A score of 0- 2 was considered low; a score of 3-4 was considered moderate; and a score of 5 was considered high.

### Data Collection and Synthesis

#### Data Extraction

After the study selection, data were extracted from eligible studies. The following data were extracted: study characteristics (author, year of publication, country, aim, types of mHealth apps used, mode of delivery, length of study/number of sessions, study design, and sample size); outcomes of each study; and main findings related to the research question of this systematic review. One reviewer (OA) performed the data extraction, and the research team checked the accuracy of the extracted data.

#### Data Synthesis

The diversity of measures and outcomes identified in the eligible studies did not allow for quantitative data synthesis; therefore, a narrative synthesis was conducted. The 9 intervention functions of the BCW [15] were used to classify intervention strategies to help inform future attempts to design interventions.

Each intervention was categorized as performing one or more of the 9 functions [22]. Definitions of the intervention functions are listed in Textbox 1.

**Definitions of intervention functions in the behavior change wheel.**

**Education**

- Increasing knowledge or understanding

**Persuasion**

- Using communication to induce positive or negative feelings or stimulate action

**Incentivization**

- Creating an expectation of reward

**Coercion**

- Creating an expectation of punishment or cost

**Training**

- Imparting skills

**Restriction**

- Using rules to reduce the opportunity to engage in the target behavior (or to increase the target behavior by reducing the opportunity to engage in competing behaviors)

**Environmental restructuring**

- Changing the physical or social context

**Modeling**

- Providing an example for people to aspire to or imitate

**Enablement**

- Increasing means or reducing barriers to increase capability or opportunity beyond environmental restructuring

## Results

### Study Selection

**Figure 2:**
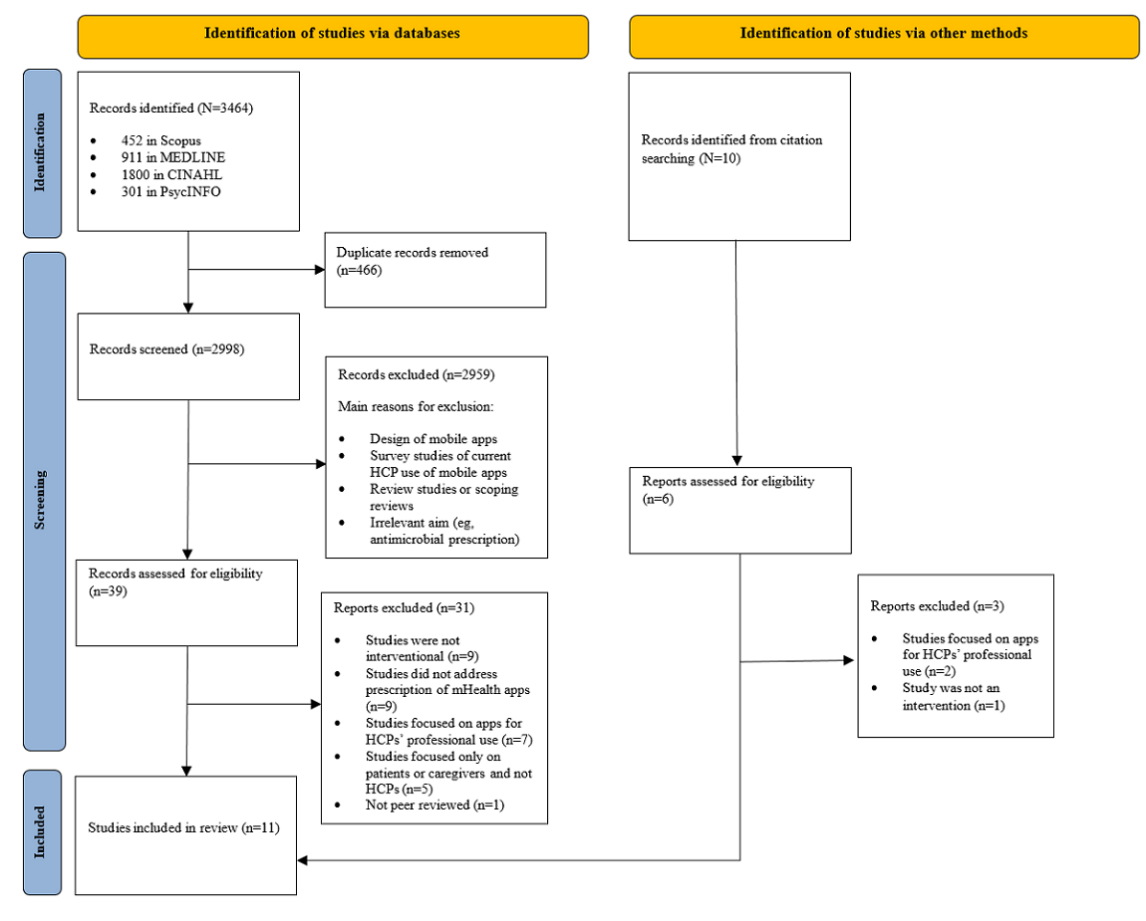
Study selection flow diagram based on the PRISMA (Preferred Reporting Item for Systematic Reviews and Meta-Analyses) guidelines. HCP: health care professional; mHealth: mobile health.

The search strategy retrieved 3464 records. Of these, 466 (13.6%) studies were duplicates and were removed, leaving 2998 (86.5%) studies for screening. The screening of titles and abstracts excluded 2959 (98.7%) studies. Therefore, 39 (1.3%) studies were eligible for full-text screening. Another 10 records were identified through citation searching, and 6 (60%) of these were included for full-text screening. Full-text screening of the 45 studies yielded a total of 11 (24%) studies in the final review. The study selection process and the reasons for exclusion are shown in Figure 2.

### Characteristics of the Included Studies

The studies were published between 2015 and 2020 and were performed in the United States (6/11, 54%), Australia (2/11, 18%), the Netherlands (1/11, 9%), Catalonia (1/11, 9%), and the United Kingdom (1/11, 9%). All the included studies contained an interventional component. Of the total 11 studies, 6 (54%) were pretest-posttest design studies with no control group; 3 (27%) were mixed methods studies; 1 (9%) was a usability study; and 1 (9%) was a qualitative description study.

The studies varied regarding study participants; some were focused on HCPs in primary care settings [23-25], whereas others targeted specific specialties, such as dietitians [26,27], behavioral health providers [28,29], providers in weight management clinics [30], clinical nutrition and physician assistant students [31], or interdisciplinary groups [32,33]. Most studies (8/11, 73%) had a sample size ranging from 5 to 40 HCPs, apart from 3 (27.3%) studies that reported the results of interventions conducted over multiple years or many training sessions in which the sample size ranged from 78 to 760 [28,29,31]. The functions of the mHealth apps used in these studies included weight management [27,30] or diet and activity tracking [26,31]. A total of 45% (5/11) of the studies used a list of approved apps for a range of health conditions [23,25,28,29,32], and 9% (1/11) used an app for chronic obstructive pulmonary disease (COPD) [33]. An overview of the characteristics of the individual studies included in the systematic review is provided in Multimedia Appendix 2 [23-33].

The most common changes resulting from the intervention were self-reported changes in knowledge, confidence, or self-efficacy [28,30-33]. Only 1 (1/11, 9%) study reported HCPs’ intention to use or recommend the app [33], and 1 (1/11, 9%) study evaluated app acceptance and use in dietetic care [27]. However, the outcomes were objectively estimated using the change in the number of apps prescribed in 36% (4/11) studies [23-25,32].

### Quality of the Evidence and Risk of Bias in the Included Studies

Using the National Institute of Health quality assessment tool, the studies by Armstrong et al [28,29], Byambasuren et al [25], Chen et al [26], and Rodder et al [31] were assessed as having a moderate risk of bias. The study by Al-Lami et al [30] was assessed as having a high risk of bias owing to a lack of clarity around several categories that were not reported, particularly regarding the selection and eligibility of participants. In all studies (6/11, 54%), it was difficult to determine whether the researchers were blinded to the intervention. An additional limitation of many of the included studies was their small sample size. Of the 11 studies, this was particularly the case for 2 studies (18%): one with 5 participants [26] and the other with 6 [30]. Most studies (5/6, 83%) lacked power calculations to accommodate the consequences of participants dropping out, thus resulting in missing values in the postintervention measurements, with the exception of Byambasuren et al [25]. The studies by Armstrong et al [28,29] and Byambasuren et al [25] did not present *P* values to compare outcome measures at pretest and posttest. The details of the quality assessment are presented in Multimedia Appendix 3 [23-33].

Using the MMAT, the qualitative description study was deemed high quality [27]. Out of the 3 mixed methods studies, 1 (33%) was judged to be of low quality [27] and 2 (67%) were of moderate quality [24,33]. The independent assessment of their quantitative and qualitative components lowered the methodological quality of all mixed methods studies. The quantitative study was deemed to be of low quality [32] owing to concerns regarding the poor description of patient selection and the high nonresponse rate. However, all studies clearly stated the objectives, interventions, and outcomes. The follow-up periods were generally short (immediately after the intervention up to 4 months after the intervention). More details on the quality assessments for each study are shown in Multimedia Appendix 3.

### Effectiveness of Interventions

The results reported by the interventions included were generally positive, with improvements being seen in several outcomes such as changes in current practices, increased knowledge, or improved self-efficacy. Although the levels of significance for the included outcomes varied, only 1 (9.1%) study found that the interventions had nonsignificant results [27].

#### Changes in Practice

A total of 54% (6/11) of studies reported changes in HCP practice following the intervention. Byambasuren et al [25] reported effective changes in GPs’ prescriptions of mHealth apps. Although the study did not report a *P* value for the number of prescriptions at pretest and posttest, the number of apps recommended per GP per fortnight increased from 1.7 to 4.1. The use of videos had no significant impact on the number of app prescriptions. Similarly, Makhni et al [32] reported the results of an 8-week trial; users at the 5 clinical sites prescribed more than 2000 apps; this exceeded the adoption targets, which was set at 100 health apps. In the study by Segui et al [23], the use of the AppSault platform to prescribe apps was reported. A total of 32 doctors made 79 app recommendations to patients, which represented 79% of the recommendations compared with what was expected during the pilot design [23]. This increase in the percentage of platform use was seen as a successful change in current practice. The staff use of apps was reported in the study by Hoffman et al [24], but it was self-reported through questionnaires. Clinical staff were receptive to apps, with 83% (19/23) incorporating behavioral health apps into their clinical work, and 25% (5/20) introducing apps to patients up to 50% of the time.

In total, 2 (18%) studies measured changes in practice using qualitative methods. First, the intervention by Korpershoek et al [33] measured the feasibility of using the Copilot app and reported HCPs’ high satisfaction and high levels of interest in the app. They also believed that the app was user-friendly and relevant to daily practice and that it fit well within the organizational culture. Second, Barnett et al [27] reported the myPace app’s acceptance among HCPs who were positive about the app. However, the uptake and recommendation of the myPace app were lower than expected.

#### Change in Knowledge

Of the 11 studies, 3 (27%) studies reported changes in the knowledge of HCPs about mHealth apps. The core competency training designed and delivered by Armstrong et al [28,29] was successful in transferring the knowledge of the enrolled clinicians. One of these studies reported the results of 3 years of the training program [28]. There was a 28.96% increase in HCPs’ self-reported knowledge of the use of mHealth in clinical care when comparing pretraining measurements (mean 2.97, SD 1.07; n=537) with posttraining measurements (mean 4.31, SD 0.76; n=537). The other study reported the results of 1 year of training and showed that the number of HCPs who rated their overall knowledge of the use of mHealth in clinical care as good or excellent before training increased from 34% (67/199) to 93% (185/199) after the training [29]. The intervention of Al-Lami et al [30] consisted of providing HCPs with a list of evaluated apps to make recommendations from, educating them about the efficacy of using mHealth apps in weight management and training them in the use of apps’ critical appraisal tools. A significant knowledge increase was reported (*P*=.02).

#### Changes in Confidence and Self-efficacy

In the remaining 2 studies, the curriculum expansion to enable physician assistant (PA) and clinical nutrition (CN) students to use mHealth apps in clinical care yielded increased self-reported confidence in their skills from pretest to posttest (*P*≤.001) [31]. The findings were supported by students’ Objective Structured Clinical Examination (OSCE) scores, which showed that both PA and CN students effectively taught standardized patients to use mobile apps for disease management. In the intervention by Chen et al [26], dietitians rated their self-efficacy before and after completing an educational and skill-training session on apps and after receiving 12 weeks of real-world experience using mHealth apps in their practice. A significant improvement in dietitians’ overall self-efficacy with mHealth apps was reported (ANOVA *F*_2, 12_=7.0; *P*=.01).

### Intervention Functions

The included studies were analyzed using the BCW framework. By doing so, the framework allowed an examination of which intervention functions are most commonly applied in the context of mHealth app prescriptions. Given that some strategies can be classified as falling into >1 category, that is, multiple intervention functions, it was difficult to link outcomes to a single intervention function. Therefore, the following sections report how the intervention strategies fit within the BCW’s 9 intervention functions. The intervention functions, outcomes, and main findings are reported separately in Table 1.

**Table 1:** Intervention functions adopted in each of the reviewed studies and main findings.

Author and year	Education	Persuasion	Incentivization	Coercion	Training	Restriction	Environmental restructuring	Modeling	Enablement	Main findings
Armstrong et al [28], 2018 and Armstrong et al [29], 2019^a^	7-hour CE^b^ workshop	Interactive material to allow learners to engage with the material	N/A^c^	N/A	mHealth^d^ apps used as examples for hands-on experience Interpersonal skills on how to discuss mobile apps with patients	N/A	N/A	Site champion to offer additional training	Site champion to offer support	In total, 93.7% reported that the information and skills learned from the training would be used in their clinical care [28]. In total, 95.8% reported that the information and skills learned from the training would be used in their clinical care [29].
Byambasuren et al [25], 2020	A letter with a brief description of each app and the study time frames and procedures was mailed.	Having prescription pads worked as a visual reminder or cue to prescribe apps Videos after 2 months demonstrating the content, features, and function of each app	N/A	N/A	N/A	N/A	Prescription pads were developed and given to GPs^e^ with apps that are relevant in general practice.	N/A	N/A	1324 app prescriptions were dispensed over 4 months The GPs’ confidence in prescribing apps doubled from a mean of 2 (not so confident) before the study to 4 (very confident) at the end of the study
Chen et al [26], 2019	Workshop to educate dietitians about a range of mHealth apps	Verbal persuasion about capabilities to master app use even in difficult situations	N/A	N/A	Case studies Dietitians were trained to appraise the quality of these apps	N/A	Easy Diet Diary Connect platform	Workshop facilitator is a dietitian modeling and working with apps Working with colleagues enabled social comparisons to be made	Ongoing support during 12 weeks of the intervention	A significant improvement in overall self-efficacy with using mHealth apps (ANOVA F2, 12=7.0; *P*=.01)
Rodder et al [31], 2018	Curriculum expansion to educate students on the use of mHealth	N/A	Pass the OSCE^f^ assessment	N/A	Students trained in how to evaluate apps using the SAAT^g^ appraisal tool Students trained in interpersonal skills, such as how to educate patients Students trained to download and use recommended apps using case studies	N/A	MyNetDiary and Withings Health Mate apps	Peer comparison	N/A	Confidence levels improved significantly for all survey measures, for both PA^h^ and CN^i^ students (*P*≤.001) OSCE results showed that both PA and CN students were able to download MyNetDiary (96.4%), enter food into the app (98.4%), and discuss the advantages of using the app for food tracking with patients (90.3%).
Al-Lami et al [30], 2019	Workshop to educate clinical staff about the efficacy of using mHealth apps in weight management	N/A	N/A	N/A	Training in how to use the Ped-WHAT App appraisal tool before making app recommendations		Providing HCPs^j^ with a list of evaluated apps from which to make recommendations	N/A	N/A	Provider knowledge of the use of apps significantly increased after the training (mean 1.00, SD 1.00 vs mean 1.67, SD 0.52; *t*=3.16; *P*=.025). Provider confidence in recommending apps to patients increased significantly after the training (mean 1.00, SD 0 vs mean 1.67, SD 0.52; *t*=3.16; *P*=.025)
Makhni et al [32], 2017	N/A	N/A	N/A	N/A	Personal training in the use of the RxUniverse platform	N/A	RXuniverse app-prescribing system	Demonstrating a trial process for prescribing an app	Considerations of the workflow to minimize disruption and time burdens of participants	During an 8-week trial, over 2000 apps were prescribed to all users in the 5 clinical sites Users felt that RxUniverse performed well. The group mean for the overall SUS^k^ score was 84.2, an “excellent” rating.
Hoffman et al [24], 2019	Series of staff meetings on best practices for using mental health apps within clinical care	N/A	N/A	N/A	N/A	N/A	A list of recommended apps for self-management Two EHR^l^ standardized “smart phrases” to facilitate the use of apps A guide to staff was created as to when to introduce and discuss apps with patients	N/A	N/A	In total,–82.6% incorporated BH^m^ apps into their clinical work; 25% introduced apps to patients 25% to-50% of the timeIn total, 42% expressed a need for more practice and training in using each tool within the CHA’s^n^ mobile app toolkit.
Korpershoek et al [33], 2020	HCPs were introduced to the Copilot app and the scenario for use of the app in daily practice, and the intended role of both HCPs and patients	N/A	N/A	N/A	A fictional patient scenario was given to HCPs, who were asked to conduct several tasks	N/A	Copilot app for COPD^o^ self-management	N/A	N/A	Main themes: high satisfaction, user-friendly, relevant for daily practice, app fit well within the organizational culture, high level of interest An average score of 83.8 (SD 15.1) on the SUS, indicating good usability of the app
Barnett et al [27], 2015	N/A	N/A	N/A	N/A	Personal training in the use of myPace software and how to make app recommendations to patients	N/A	MyPace app for weight loss designed to fit daily dietetic practice	N/A	N/A	The dietitians were positive and enthusiastic about the app; however, their enthusiasm did not translate into actual uptake, use, and recommendation
Segui et al [23], 2018	Instructions to train HCPs in prescribing and using the platform	Follow-up and monitoring	N/A	N/A	N/A	N/A	AppSalut platform to prescribe apps	N/A	Support by periodic follow-ups accompanied by training for doctors and solving any technical problems	A total of 32 doctors made 79 app recommendations to patients, representing 160% of doctors and 79% of recommendations compared with what was expected

^a^Studies are combined owing to similar intervention components.

^b^CE: continuing education.

^c^N/A: not applicable.

^d^mHealth: mobile health.

^e^GP: general practitioner.

^f^OSCE: Objective Structured Clinical Examination.

^g^SAAT: smartphone application appraisal tool.

^h^PA: physician assistant.

^i^CN: clinical nutrition.

^j^HCP: health care practitioner.

^k^SUS: system usability score.

^l^EHR: electronic health record.

^m^BH: behavioral health.

^n^CHA: Cambridge Health Alliance.

^o^COPD: chronic obstructive pulmonary disease.

#### Education

In total, of the 11, 9 (81.8%) studies included elements of education. Education came in the form of workshops in 4 studies [26,28-30]. The workshops covered the best practices for using mHealth apps in patient care and considerations of privacy, security, and ethical and cultural issues of using mHealth apps with patients [28,29]. The workshop in the study by Chen et al [26] educated dietitians about the range of commercially available apps. Similarly, the workshop in the study by Al-Lami et al [30] provided background information on mobile apps’ efficacy and validity in weight management therapy. It introduced a list of apps to use when making recommendations to patients. However, Hoffman et al [24] used a series of staff meetings as the mode of delivery to educate behavioral health staff about the best practices for using mHealth apps with patients. One study, which was conducted in an academic health center, also involved education through curriculum expansion and focused on mHealth apps [31].

In the study by Byambasuren et al [25], education was part of the intervention in 2 ways. A letter was mailed to each participant describing the study and containing instructions and guides on prescribing the app . The second education element was also delivered via videos showing the app’s content, features, and functions. In this study, the authors aimed to assess the impact of videos on the number of app prescriptions.

Creating guides or providing HCPs with instructions on when to introduce and discuss apps with patients were carried out in 2 studies as an educational form [23,24]. Another study used education; however, the content was tailored to the platform under testing. Koreospek et al [33] introduced the Copilot app to study participants and explained the intended use of the app for the self-monitoring of symptoms by patients with COPD. Moreover, a tutorial was given on how to use the app and perform essential functions, such as registering patients and customizing an action plan.

These educational elements of the interventions aimed to improve HCPs’ knowledge. In addition, of the 11 studies, 3 (27%) captured and reported changes in self-reported knowledge before and after the intervention [28-30]. The remaining studies measured different outcomes, such as a change in self-confidence [25,31], self-efficacy [26], or an increased number of prescriptions [25]. However, these measures were reported as the results of the intervention as a whole and were not specific to education.

#### Persuasion

A total of 45% (5/11) of the studies reported elements of persuasion. Periodic follow-ups with study participants served as a method of reminding and motivating HCPs to modify their prescribing habits [23]. Visual reminders were used in another study in 2 forms. First, videos were sent to study participants after month 2 of the intervention [25]. These videos not only were educational but also worked as a tool to remind study participants of the study. Second, the design and dissemination of prescription pads involved reminders or cues to prescribe apps.

Persuasion was reflected in both studies by Armstrong et al [28,29]. The development of educational materials involved adopting evidence-based interactive educational experiences to allow learners to engage in the material and ensure promising results regarding behavior change. In a study by Chen et al [26], persuasion was sought verbally to convince dietitians of their capabilities to prescribe mHealth apps, even in difficult situations such as short consultations.

#### Incentivization

Incentivization can be social, such as a promotion in status, or fiscal. Students’ desires to pass the OSCE exam by demonstrating their capabilities to recommend and use mHealth apps in nutrition care worked as a social incentive [31]. According to the OSCE results, PA and CN students successfully taught patients how to use mobile apps to track food intake and test blood pressure. None of the remaining interventions used strategies that fall into the incentivization category.

#### Training

A total of 8 (72.7%) interventions reported elements of training to improve HCPs’ skills when using apps. The focus and range of skills offered to HCPs varied in each study. Three interventions used case studies or scenarios to help HCPs develop and master the basic skills of using and recommending apps [26,31,33]. Training on how to use Ped-WHAT, an app appraisal tool, when making recommendations to patients and their families was critical in the intervention by Al-Lami et al [30]. Similarly, Rodder et al [31] trained students to evaluate mobile apps using a smartphone app appraisal tool.

Participants received personal training in using myPace software and were allowed to practice with it [27]. They were also allowed to provide feedback on their first impression of the software during these training sessions and were encouraged to use or recommend the app to make it a standard tool to support everyday practice. The training in the intervention by Makhni et al [32] intervention was also individualized. HCPs were instructed on how to use the RxUniverse system to make app prescriptions and were then observed to ensure successful app prescription.

The term “hands-on experience” was used in the study by Armstrong et al [28] to refer to practical experience; however, if training was involved, it was not detailed enough. Interpersonal skills were targeted in 2 interventions [28,29]. Competencies in discussing mobile apps with patients and showing an understanding of patients’ concerns about privacy and security were at the core of these interventions. Rodder et al [31] also mentioned measuring students’ communication skills in OSCE examinations. Students were asked to demonstrate skills in discussing the benefits of using apps for food tracking and blood pressure measurement.

#### Environmental Restructuring

To promote app prescription behavior among HCPs, 9 interventions contained methods of environmental restructuring. Most included studies offered technical resources to facilitate app prescriptions, such as developing an electronic platform. The study by Segui et al [23] tested the feasibility of using an app catalog named AppSault for recommending apps to patients. The apps were free to download and passed the quality control process, guaranteeing a safe and reliable environment for their use. Makhni et al [32] used RxUniverse, a digital medicine-focused care delivery platform with a library of apps chosen based on published evidence-based reviews of their efficacy and usability. Some studies provided HCPs with specialized apps and measured how they fit daily practice. The MyNetDiary and Withings Health Mate apps were provided for PA and CN students in a study by Rodder et al [31], myPace for dietitians in a study by Barnett et al [27], and Copilot for COPD self-management in a study by Korpershoek et al [33]. The Easy Diet Diary Connect platform was used to allow dietitians to track patients in a study by Chen et al [26]. Other studies have used lists of apps given to HCPs to make recommendations [24,25,30].

Environmental restructuring came in the form of EHRs’ standardized “smart phrases” to facilitate the prescription process [24].

#### Modeling

A total of 5 (45.5%) studies reported on methods of modeling [26,28,29,31,32]. The intervention by Armstrong et al [28,29] used “site champions” as onsite facilitators who offered additional training to local staff. Modeling was presented in different forms in the intervention by Chen et al [26]. First, the workshop moderator, who was also a dietitian, like the other study participants, modeled and used the app. Second, during the intervention, working with colleagues who had participated in the study and were successful in using the app enabled a social comparison to be made. Another study used modeling by comparison and competition among students or peers of each class [31]. Makhni et al [32] used a similar modeling method by demonstrating the functionality of the platform to participants who were thereafter asked to prescribe an mHealth app.

#### Enablement

Enablement was used in 5 (45.5%) studies. In 1 study, enablement came in the form of ongoing support throughout the study to reduce barriers associated with prescribing mHealth apps [26]. Support was also provided to study participants by consulting office managers and study participants about the specific operational workflows of each clinical site and the optimal implementation plan for RxUniverse at each pilot site to minimize the time burden [32]. The periodic follow-ups in the study by Segui et al [23] were accompanied by solving any technical problems, which enabled the implementation of corrective actions and extra training [23]. Recognizing site champions in Armstrong et al [28,29] offered support to sustain behavior change after the training.

#### Coercion and Restriction

None of the interventions included in this review used the intervention functions of coercion or restriction.

### Linking Interventions With the COM-B Model

We looked at the most often used intervention functions in the studies with successful outcomes to gain a better understanding of why the included intervention functions provided substantial changes. As seen in Table 1, successful interventions used a variety of intervention functions (environmental restructuring, education, and training). These intervention functions cover most components of the COM-B model, which suggests that interventions are more likely to produce effective results and make changes in current behavior if they target a wide spectrum of the COM-B model’s components. For example, the study by Chen et al [26] used all COM-B components to increase dietitians’ app use behaviors through dietitians’ education and skill training as well as environmental restructuring by providing physical app-based infrastructure. Modeling, coaching, and improving self-efficacy were also addressed. Therefore, the reported change in ratings of dietitians’ self-efficacy when using mHealth apps was significant (*P*=.01). The Tukey post hoc test revealed significantly higher post–workshop mHealth app self-efficacy ratings compared with the baseline (*P*=.02), and the ratings were sustained at 12 weeks (*P*=.01).

In addition, the intervention functions used in the ineffective study were linked to only 2 COM-B model components (physical capability and physical opportunity), suggesting a need to understand the target behavior by collecting data from multiple sources to ensure successful behavior change.

## Discussion

### Principal Findings

This review included 11 studies investigating the effects of interventions aimed at encouraging app prescription behavior among HCPs. Most studies demonstrated positive findings for outcomes such as self-confidence, knowledge of mHealth apps, and number of app prescriptions. The studies included differed widely in terms of interventions, measures of practice change, types of mHealth apps used, modes of delivery, and study settings.

A broad range of interventions, all related to methods for enhancing mHealth app adoption, specifically mHealth prescribing, was covered in this review, including education (workshops, class lectures, individual sessions with a dietitian or research team, videos, or toolkits), persuasion (reminders or verbally about capabilities), incentivization (expectation to pass the course), training (case studies or scenarios or app appraisal tools), modeling (site champions or observing peers), enablement (support), and environmental restructuring (lists of apps, technological systems, time, and resources).

### Comparisons With Other Works

More than half of the interventions included in this review had training (8/11, 72.7%) or educational (9/11, 81.8%) functions that targeted HCPs’ capabilities (physical or psychological). A lack of knowledge and awareness of available apps are major barriers reported in the literature [34-36]. A study reported that HCPs consider the lack of knowledge of available apps that have proven their effectiveness in improving patient health outcomes an important barrier to prescribing [35]. This confirms the urgent need to provide training programs or educational sessions regarding the available mHealth apps that support patient self-management of long-term conditions.

However, the terms training and education are often used interchangeably. Some studies reported training as part of the intervention, but these studies included only elements of imparting knowledge and understanding, not skill development [24,28]. One possible explanation is the lack of attention to different types of training in behavior change interventions. Hence, there is difficulty in perceiving what training might entail. Therefore, it is vital to distinguish between the 2 terms. This overlap has also been reported between other intervention functions. Another study reported the existence of some overlap when providing education to participants because it serves a persuasion function at the same time [37]. This is mainly because education may induce positive feelings toward app prescription.

The lack of coercion (defined as “creating expectation of punishment or cost”) may be because it was not deemed a suitable approach for improving self-efficacy or confidence in app prescriptions or it may have been deemed counterproductive when trying to create positive attitudes or encourage app prescriptions. Furthermore, it is impractical to penalize HCPs for not prescribing mHealth apps.

None of the interventions adopted restrictions such as rules to increase HCP app prescriptions or decrease any competing behavior. The absence of regulations that ensure apps’ highest quality and accuracy and the lack of data validity and reliability of existing apps keep HCPs from prescribing apps to their patients [38,39]. To compensate, some interventions have provided study participants with a list of trustworthy apps to use when making app recommendations to patients. Other interventions adopted digital solutions such as building software or electronic systems containing approved apps. Removing such barriers by providing practical resources is a form of environmental restructuring.

Minimizing the disruption of HCPs’ time is a form of enablement reported in only one intervention [32]. HCPs’ concerns about time to discuss and instruct patients on how to use apps were reported as barriers to app prescription. In a pilot study, participants were concerned that recommending apps to patients would lengthen the duration of consultations [23]. In France, a qualitative study presented a theme after interviews with GPs about “Doctor Protection,” which mainly introduced concerns about increased workload and prescriptions of apps as an additional task [40]. One significant distinction between apps and medications is that many drugs can be prescribed with simple directions, whereas an app may require more specific instructions.

One way to minimize physicians’ workloads is to integrate and synchronize health information produced from mHealth apps to patients’ EHRs. By doing this, the physician’s ability to access patient data is centralized. In an acceptability and feasibility study that examined integrating patient data generated from smartphones into EHRs [41], clinicians reported that by using the graph feature, they could evaluate longitudinal data during consultations, which was quick and easy. When compared with retrieving information by recording histories, this was thought to be a possible time saver. Furthermore, this approach provided an accurate reflection of disease changes and treatment responses.

### Strengths and Limitations of the Study

This review has several strengths and limitations. This is the first review that addresses interventions to improve HCPs’ confidence and capabilities to prescribe or recommend mHealth apps to patients. We consider the included studies to be a complete set of studies from 2008 to 2022. The studies were sourced from a variety of electronic databases, with the reference lists of the included papers checked for potentially relevant studies. This systematic review used a robust methodology that included screening all the studies for relevance by 2 independent reviewers.

However, the number of studies included in this review was limited, and the findings depended on the quality of the included studies. Of the 11 studies, 3 (27.3%) studies were found to be at high risk of bias, 7 (63.6%) at moderate risk of bias, and only 1 (9.1%) at low risk of bias (see Appendix 3). A total of 6 (54.5%) studies were pretest-posttest design interventions; these are known for their methodological issues such as selection bias and short durations, which do not make it possible to determine whether the intervention is effective and sustainable [42]. Furthermore, 7 (63.6%) studies used self-reported data to reflect possible changes in behaviors. With self-reported outcomes, it is impossible to tell whether the reported change in knowledge or practice is owing to response-shift bias or an actual adoption of the targeted behaviors. This emphasizes the importance of using objective measures instead. Objective measures such as the number of app prescriptions and OSCE scores were reported in 4 (36.4%) studies. However, the assessment of behavior changes or intentions to change app prescription behaviors cannot always be performed using objective measures. This is the case, in particular with interventions that lack technical systems to track the changes in the number of prescriptions before and after the intervention. Combining objective and self-reported measures could bring more insight and different perspectives to the study findings.

### Unanswered Questions and Future Research

Several gaps in the research were identified in this review. Coercion and restriction were not reported in any of the interventions included; however, this may be because they were not deemed appropriate approaches for changing app prescription behaviors in this population. The impact of other forms of intervention functions not used in any of the interventions reviewed could be explored, such as the use of incentives (financial or nonfinancial) that, if appropriately applied and supported with other intervention functions, could potentially make an impact and encourage HCPs to prescribe apps [43]. Evidence on the acceptability and impact of such programs in the context of mHealth is lacking. This can be answered with future studies.

High-quality studies with adequate sample sizes and longer study periods are now essential for detecting differences in app prescription behaviors. Future interventions could adopt theoretical frameworks and behavior change frameworks to systematically understand HCPs behavior toward the prescription of mHealth apps.

The COM-B model, as part of the BCW, is a useful tool to make behavioral diagnoses and identify what needs to change [22]. Evidence from successful interventions, interviews, or surveys with HCPs about what motivates or limits their mHealth app prescription behaviors can provide sufficient information to understand the sources of those behaviors. Therefore, future interventions can address the target behavior (app prescriptions) and its influencing factors.

### Conclusions

This study identified interventions aimed at improving HCPs’ app-prescribing behaviors. On the basis of the BCW, environmental restructuring and education were the most frequently used intervention functions in the included studies, followed by training. The findings of this study provide evidence that combining elements of training, education, and environmental restructuring is more likely to produce effective changes in HCPs’ behavior toward app prescribing.

## Appendix

Multimedia Appendix 1 Results of the search strategies used in the MEDLINE and CINAHL databases.

Multimedia Appendix 2 Characteristics of individual studies included in the systematic review.

Multimedia Appendix 3 Risk of bias assessment of included studies.

## Abbreviations

BCW: behavior change wheel
CN: clinical nutrition
COM-B: capability, opportunity, motivation, and behavior
COPD: Chronic Obstructive Pulmonary Disease
EHRs: electronic health records
GPs: general practitioners
MMAT: mixed methods appraisal tool
OSCE: Objective Structured Clinical Examination
PA: physician assistant
PRISMA: Preferred Reporting Items for Systematic Reviews and Meta-analyses
