# Supplementary material for: Interventions Aimed at Enhancing Health Care Providers’ Behavior Toward the Prescription of Mobile Health Apps: Systematic Review
Source: JMIR Mhealth Uhealth. 2023 Feb 27;11:e43561. doi: 10.2196/43561 (PMC10012012; doi:10.2196/43561)
Supplement: Multimedia Appendix 1 [file mhealth_v11i1e43561_app1.docx]

# Multimedia Appendix 1

Results of the search strategies used in MEDLINE and CINAHL

**MEDLINE**

| # | Searches | Results |
| --- | --- | --- |
| 1 | "Healthcare provider".mp. or Health Personnel/ | 62,072 |
| 2 | physician.mp. or Physicians/ | 325,364 |
| 3 | General Practitioners/ or General practitioner*.mp. | 53,082 |
| 4 | GP.mp. | 42,076 |
| 5 | Resident*.mp. | 198,003 |
| 6 | Clinician*.mp. | 238,683 |
| 7 | dentist.mp. or Dentists/ | 37,782 |
| 8 | dietician*.mp. | 1,829 |
| 9 | dietitian*.mp. or Nutritionists/ | 7,174 |
| 10 | Physical Therapists/ or physiotherapist*.mp. | 9,482 |
| 11 | cardiologist*.mp. or Cardiologists/ | 15,222 |
| 12 | gynaecologist*.mp. | 3,622 |
| 13 | Ophthalmologists/ or ophthalmologist*.mp. | 13,940 |
| 14 | psychiatrist*.mp. | 24,035 |
| 15 | 1 or 2 or 3 or 4 or 5 or 6 or 7 or 8 or 9 or 10 or 11 or 12 or 13 or 14 | 953,592 |
| 16 | interven*.mp. | 1,128,911 |
| 17 | behav* change.mp. | 20,911 |
| 18 | behav* interv*.mp. | 11,215 |
| 19 | Persuasive Communication/ or persuas*.mp. | 7,637 |
| 20 | Reward/ or reward*.mp. | 59,524 |
| 21 | Curriculum/ or Educat*.mp. | 1,059,597 |
| 22 | Training.mp. | 453,456 |
| 23 | Workshop.mp. or Education/ | 47,880 |
| 24 | Tutorial.mp. | 5,436 |
| 25 | 16 or 17 or 18 or 19 or 20 or 21 or 22 or 23 or 24 | 2,360,569 |
| 26 | Prescri*.mp. or Prescriptions/ | 235,392 |
| 27 | Recommend*.mp. | 677,474 |
| 28 | Adopt*.mp. | 25,7671 |
| 29 | Suggest*.mp. | 4,056,464 |
| 30 | Discuss*.mp. | 1,423,008 |
| 31 | 26 or 27 or 28 or 29 or 30 | 6,135,683 |
| 32 | "Mobile Applications".mp. or Mobile Applications/ or Cell Phone/ | 19,669 |
| 33 | Mobile health.mp. | 8,553 |
| 34 | mhealth.mp. | 5,086 |
| 35 | m health.mp. | 612 |
| 36 | ((mobile or phone or telephone) adj (app? or application?)).ti,ab,kf. | 6,272 |
| 37 | health app.mp. | 297 |
| 38 | m-health app.mp. | 10 |
| 39 | mobile medical applications.mp. | 37 |
| 40 | "mobile medical application".mp. | 14 |
| 41 | smartphone app*.mp. | 3,757 |
| 42 | Medical app.mp. | 56 |
| 43 | digital health.mp. | 3,383 |
| 44 | mobile phone.mp. or Cell Phone/ | 12,948 |
| 45 | Digital therapeutic.mp. | 74 |
| 46 | smartphone application*.mp. | 2,303 |
| 47 | 32 or 33 or 34 or 35 or 36 or 37 or 38 or 39 or 40 or 41 or 42 or 43 or 44 or 45 or 46 | 35,024 |
| 48 | 15 and 25 and 31 and 47 | 956 |
| 49 | limit 48 to yr="2008 - 2022" | 933 |
| 50 | limit 49 to English language | 911 |

**CINAHL**

|  | Searches | Results |
| --- | --- | --- |
| 1. | Mobile Application* OR Cell Phone OR Mobile phone OR smartphone OR mHealth OR Mobile health OR Digital Health OR Digital therapeutic OR Health apps OR m-health app OR medical app OR Mobile Medical App OR smartphone application | 49,914 |
| 2. | Prescri* OR Recommend* OR Adopt* OR Suggest OR use | 1,974,329 |
| 3. | Health Personnel OR GP OR General Practitioner* OR Physician OR Clinician OR "Healthcare worker" OR "Healthcare provider" OR "Healthcare professional" OR resident* OR dentist* OR dietician* OR dietitian OR physical therapist* OR physiotherapist* OR cardiologist* OR gynaecologist* OR ophthalmologist* OR psychiatrist* | 702,128 |
| 4. | Interven* OR Training OR behave* change OR behav* interv* OR Persuas* OR reward OR curriculum OR educat* OR Workshop OR tutorial | 1,517,168 |
| 5. | S1 AND S2 AND S3 AND S4 | 1,800 |
