# Supplementary material for: Interventions Aimed at Enhancing Health Care Providers’ Behavior Toward the Prescription of Mobile Health Apps: Systematic Review
Source: JMIR Mhealth Uhealth. 2023 Feb 27;11:e43561. doi: 10.2196/43561 (PMC10012012; doi:10.2196/43561)
Supplement: Multimedia Appendix 2 [file mhealth_v11i1e43561_app2.docx]

**Multimedia Appendix 2:** Characteristics of individual studies included in the systematic review

| **(Author, Year)** | **Study design** | **Country** | **Study setting** | **Focus** | **Number of sessions / Length of the study** | **Mode of delivery** | **mHealth app/s used** | **Data collection method** | **Sample size** |
| --- | --- | --- | --- | --- | --- | --- | --- | --- | --- |
| **Armstrong et al, 2018** [28] | Pre- post study with no control group | USA | Military and VA hospitals | Development and evaluation of a  competency-based training programme on the integration of mobile  health into clinical settings | 20 one-day workshops | Face-to-face | A platform with range of apps for veterans’ health and mental health (https://mobile.va.gov/appstore) | Survey | 760 |
| **Armstrong et al, 2019** [29] | Pre- post study with no control group | USA | Military and VA hospitals | Effectiveness of a competency-based training program to train  providers in best practices for mobile health in clinical care | 8 one-day, 7-hour continuing education training session | Face-to-face | A platform with range of apps for veterans’ health and mental health (https://mobile.va.gov/appstore) | Survey | 252 |
| **Byambasuren et al, 2020** [25] | Pre- post study with no control group | Australia | Australian general practice setting | Effectiveness of an implementation intervention to increase uptake of app prescription | 4 months | Distance via mail and e-mail | - This Way Up: Managing Depression  - Tät – Pelvic floor exercises  - Lose-It!  FitNow Inc  - CBT-i Coach  - Smiling Mind  - Quit Now: My QuitBuddy | Prescription pads, survey, and interviews | 40 |
| **Chen et al, 2019**[26] | Pre- post study with no control group | Australia | Accredited Practicing Dietitians (APDs) working in private practice setting | Educational intervention to integrate apps into dietetic practice and improve dietitians' perceived self-efficacy toward using mHealth apps in patient nutrition care | 4-hour educational and skills training workshop | Face-to-face | Easy Diet Diary app (Xyris Software Australia Pty Ltd) | Survey | 5 |
| **Rodder et al, 2018** [31] | Pre- post study with no control group | USA | Departments of Clinical Nutrition and Physician Assistant Studies, UT Southwestern Medical Center | Effectiveness of curriculum expansion on physician assistant (PA) and clinical nutrition (CN) students' confidence in their ability to use mobile apps to provide lifestyle counselling in weight loss, diabetes, and heart disease | During spring semesters in 2015 through 2018 | Not reported | - MyNetDiary  - Withings Health Mate | Survey, objectively structured examination station scores (OSCE) | **PA**  (n = 173)  **CN**  (n = 78) students |
| **Al- Lami et al, 2020**[30] | Pre- post study with no control group | USA | Weight management clinic for children and adolescents at a university teaching hospital | Assess the impact of an educational in-service intervention on the provider's knowledge and self-efficacy in the use of the app assessment tool | The training lasted for 1 hour | Not reported | Weight management apps | Survey | 6 |
| **Makhni et al, 2017** [32] | Usability study | USA | 2 primary care clinics  1 pulmonology clinic  1 gastroenterology clinic  1 cardiology clinic | Assess the usability and adoption of RxUniverse to facilitate curation and dissemination of mHealth apps | 5–7 minutes/ 8-week pilot period | Not reported | A platform with a range of apps (http://rxuniverse.com/):  - Health education  - Patient surveys  -Remote monitoring  - Integrated wearables  - Telemedicine  - Appointment scheduling  - Social networks  - Clinical trials | Number of apps prescribed from the RxUniverse, Questionnaire | 40 |
| **Hoffman et al, 2019** [24] | Mixed-methods study | USA | Primary care (32 behavioural health care service at Cambridge Health Alliance) | Test the feasibility of using mental health applications to augment integrated primary care services | Initial pilot: 2 months  staff training and dissemination: a year | Not reported | Mental health apps: Breathe2Relax  CBT-iCoach  Mindfulness coach  Mood tools  T2 Mood Tracker  Self-help for anxiety management  Quit guide | Survey | 32 |
| **Korpershoek et al, 2020** [33] | Mixed-methods study | Netherlands | 5 primary, 7 secondary, and 2 tertiary care settings | Evaluate the perceived feasibility of the copilot app in the daily practice of HCPs | A single, 1-hour session | Not reported | COPD Self-Management app (Copilot) | Observations and the Think Aloud Method, semi-structured interview and questionnaire | 14 |
| **Barnett et al, 2015** [27] | Qualitative study | UK | Working in either freelance practice or in the NHS | To report on the design, development and adoption of MyPace app | Not reported | Face-to-face | Weight management app (MyPace) | Coaching think aloud and semi-structured interviews | 20 |
| **Segui et al, 2018**[23] | Mixed methods study | Catalonia | Primary care | Test, in a real, controlled environment, the implementation of AppSalut | 6 months (June 1 to November 30, 2017) | Not reported | A platform with a range of apps:  AsmaProcare for Asthma, ExpertSalud for Chronicity, and Sideal for alcohol consumption | Data from the AppSalut system and questionnaire | 32 |
