# Supplementary material for: Interventions Aimed at Enhancing Health Care Providers’ Behavior Toward the Prescription of Mobile Health Apps: Systematic Review
Source: JMIR Mhealth Uhealth. 2023 Feb 27;11:e43561. doi: 10.2196/43561 (PMC10012012; doi:10.2196/43561)
Supplement: Multimedia Appendix 3 [file mhealth_v11i1e43561_app3.docx]

# Multimedia Appendix 3

Table 1: Risk of bias assessment using the NIH quality assessment tool for before and after (pre-post) studies with no control group

|  | Objective clearly stated | Eligibility criteria described | Representative patient population | All eligible participants enrolled in study | Sufficient sample size | Intervention described | Outcome measures specified | Outcome assessors blinded | Loss to follow-up | Statistical analysis of outcome measures before and after intervention | Interrupted time-series design | Individual data used for group-level effects | Total score | Overall quality of the study |
| --- | --- | --- | --- | --- | --- | --- | --- | --- | --- | --- | --- | --- | --- | --- |
| Armstrong et al, 2018 [28] | ● | ● | ● | ● | ● | ● | ● | ○ | ● | ● | ● | ● | 7 | Fair |
| Armstrong et al, 2019 [29] | ● | ● | ● | ● | ● | ● | ● | ○ | ● | ● | ● | ● | 7 | Fair |
| Byambasuren etal, 2020 [25] | ● | ● | ● | ● | ● | ● | ● | ○ | ● | ● | ● | ● | 9 | Fair |
| Chen et al, 2019 [26] | ● | ● | ● | ● | ● | ● | ● | ○ | ● | ● | ● | ● | 7 | Fair |
| Rodder et al, 2018 [31] | ● | ● | ● | ● | ● | ● | ● | ○ | ● | ● | ● | ● | 8 | Fair |
| Al-Lami et al, 2020 [30] | ● | ● | ● | ● | ● | ● | ● | ○ | ● | ● | ● | ● | 4 | Poor |

●= Yes, ●= No, ●= Cannot determine, ●= Not applicable, ○= Not reported, Total Score: Number of yes

Table 2: Risk of bias assessment using the MMAT quality assessment tool

|  | 1. Qualitative | | | | | 4. Quantitative descriptive | | | | | 5. Mixed methods | | | | | Quality of the study |
| --- | --- | --- | --- | --- | --- | --- | --- | --- | --- | --- | --- | --- | --- | --- | --- | --- |
| (Author, Year) | **1.1** | **1.2** | **1.3** | **1.4** | **1.5** | **4.1** | **4.2** | **4.3** | **4.4** | **4.5** | **5.1** | **5.2** | **5.3** | **5.4** | **5.5** |  |
| Barnett et al, 2015 [27] | Yes | Yes | Yes | Yes | Yes |  |  |  |  |  |  |  |  |  |  | High |
| Hoffman et al, 2019 [24] |  |  |  |  |  |  |  |  |  |  | No | Yes | Yes | Yes | No | Moderate |
| Korpershoek et al, 2020 [33] |  |  |  |  |  |  |  |  |  |  | Yes | Yes | Yes | Yes | No | Moderate |
| Makhni et al, 2017 [32] |  |  |  |  |  | Can't tell | No | Yes | No | Yes |  |  |  |  |  | Low |
| Segui et al, 2018[23] |  |  |  |  |  |  |  |  |  |  | No | No | No | Can’t Tell | No | Low |
